# Bleeding giant pseudoaneurysm non-visualized on arterial phase imaging: Endoscopic ultrasound-guided angioembolization to the rescue

**DOI:** 10.1055/a-2081-8158

**Published:** 2023-05-26

**Authors:** Jayanta Samanta, Jahnvi Dhar, Mithu Bhowmick, Ashutosh Ishan, Antriksh Kumar, Pankaj Gupta, Saroj Kant Sinha

**Affiliations:** 1 Department of Gastroenterology, Postgraduate Institute of Medical Education and Research, Chandigarh, India; 2 Department of Radiodiagnosis and Imaging, Postgraduate Institute of Medical Education and Research, Chandigarh, India

A 51-year-old man, with known diabetes and coronary artery disease and a history of acute pancreatitis, presented with melena for 1 day. Investigations revealed anemia (hemoglobin 6.8 gm/dl) with tachycardia. After initial resuscitation, computed tomography (CT) angiography was done. In the arterial phase, no extravasation or aneurysm was noted ( Fig. 1 ). However, in the venous phase, contrast filling was noted with a giant pseudoaneurysm measuring 7.2 × 6.2 × 9.7 cm, likely arising from the splenic vessel ( Fig. 2 ). The patient, being a poor candidate for radiological endovascular therapy (non-visualization on arterial phase, narrow neck) as well as surgery (multiple comorbidities), was planned for endoscopic ultrasound (EUS)-guided angioembolization. EUS-guided localization of the aneurysm was done and Doppler showed turbulent blood flow in the giant pseudoaneurysm ( Fig. 3 ). It was punctured with a 19-G needle (EZ Shot3 Plus; Olympus Medical, Tokyo, Japan) and blood aspirated to confirm the position. After flushing the needle with saline, four Nester coils (20 mm × 14 cm) were deployed one after the other. Using this coil-complex as a scaffold, 4 ml of cyanoacrylate glue was injected. The coil-glue cast formed caused thrombosis of the blood contents, which gradually increased in size and slowed the intravascular turbulence ( Fig. 4 **)** . On further observation for another 1 minute, the whole aneurysm showed formation of an echogenic thrombus with minimal flow ( Video 1 ). A repeat EUS 48 hours later showed complete obliteration of the pseudoaneurysm with no flow ( Fig. 5 ), and CT revealed coil artifacts with no filling in the venous phase. At the 1-year follow-up, the patient was doing fine with no further bleeding episodes.

**Fig. 1:**
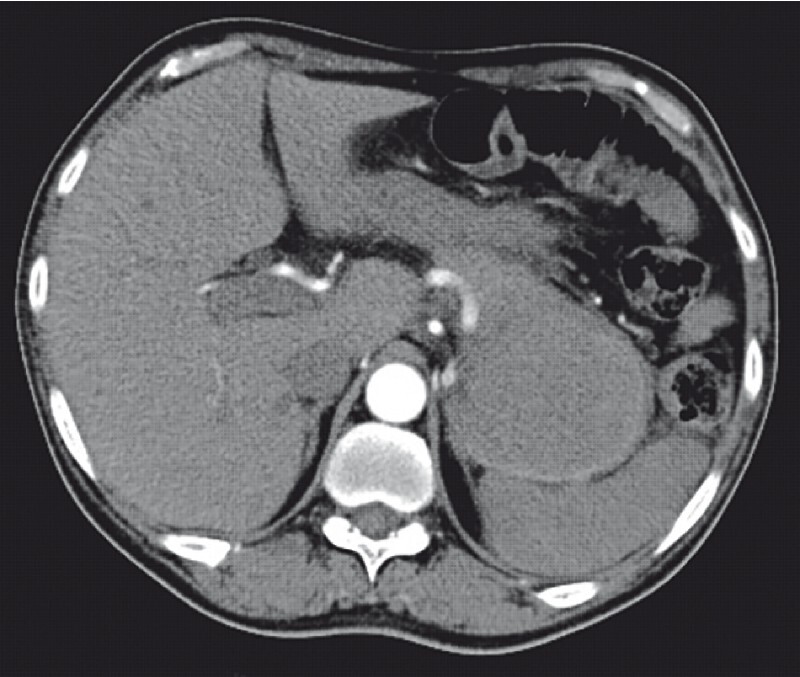
Contrast-enhanced computed tomography (CECT) of the abdomen and computed tomography angiography (CTA) (arterial phase), which revealed no contrast extravasation or pseudoaneurysm.

**Fig. 2:**
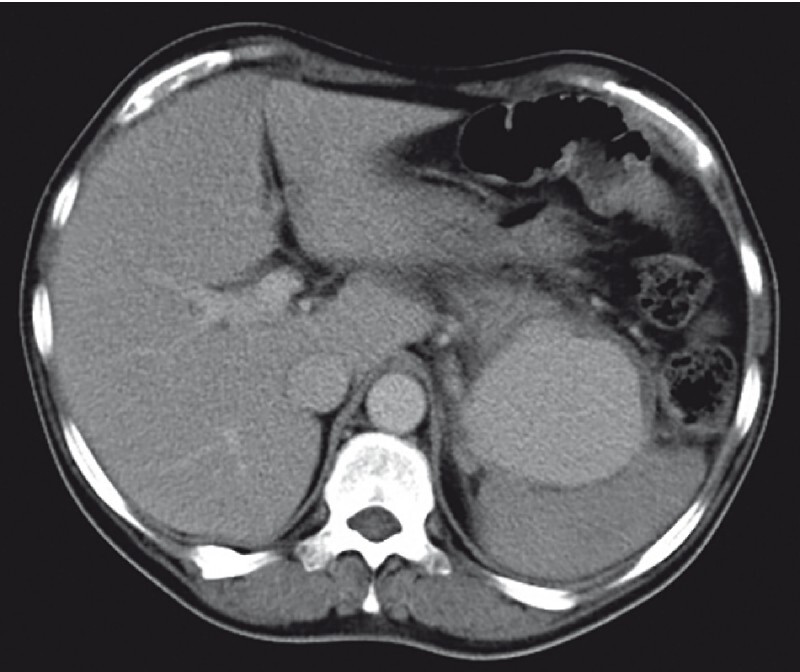
Abdominal CECT and CT angiography (venous phase) revealed contrast filling with a giant pseudoaneurysm measuring 7.2 × 6.2 × 9.7 cm, likely arising from the splenic vessel.

**Fig. 3:**
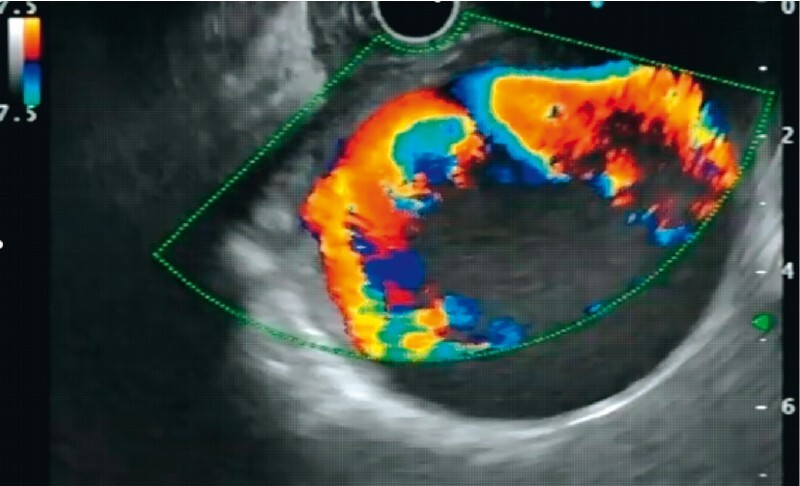
Endoscopic ultrasound (EUS) localization of the giant pseudoaneurysm (mimicking a pseudocyst), with Doppler showing turbulent blood flow.

**Fig. 4:**
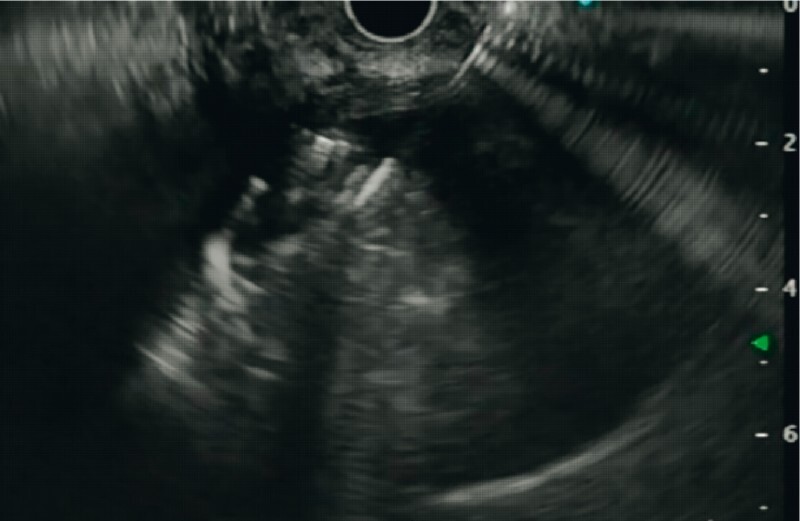
EUS-guided deployment of coil and glue to form a cast.

**Video 1** Video showing endoscopic ultrasound (EUS)-guided angioembolization of a giant pseudoaneurysm (arising from the splenic vessel), visible only in the venous phase, with coil and cyanoacrylate glue, leading to complete obliteration.


**Fig. 5:**
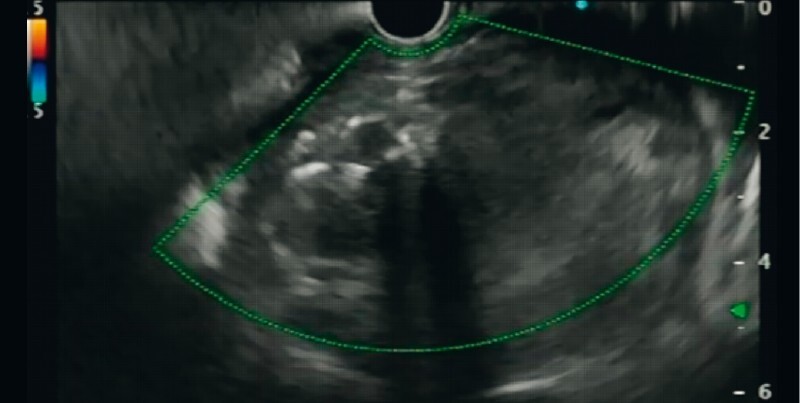
Repeat EUS after 48 hours revealed complete obliteration of the pseudoaneurysm with coil-glue complex with no flow noted on Doppler.

Thus, this case demonstrates that packing the whole aneurysm with coils is not always mandatory. Careful observation of the flow dynamics during the procedure can help assess the requirement of coils in real time and thus lower the number of coils required.

Endoscopy_UCTN_Code_TTT_1AS_2AD

## Notes

Endoscopy E-Videos

https://eref.thieme.de/e-videos
*E-Videos* is an open access online section of the journal *Endoscopy* , reporting on interesting cases and new techniques in gastroenterological endoscopy. All papers include a high-quality video and are published with a Creative Commons CC-BY license. Endoscopy E-Videos qualify for HINARI discounts and waivers and eligibility is automatically checked during the submission process. We grant 100% waivers to articles whose corresponding authors are based in Group A countries and 50% waivers to those who are based in Group B countries as classified by Research4Life (see: https://www.research4life.org/access/eligibility/ ). This section has its own submission website at https://mc.manuscriptcentral.com/e-videos

